# CSF-venous fistulae—An underrecognized cause of superficial siderosis

**DOI:** 10.1177/15910199251339542

**Published:** 2025-05-05

**Authors:** Emanuele Orru’, Christopher Adereti, Neil V Patel, Timo Krings, Jonathan Pace

**Affiliations:** 1 Division of Neurointerventional Radiology, 2094 Lahey Hospital & Medical Center – Beth Israel Lahey Health, UMass Chan Medical School, Boston, MA, USA; 2 Division of Neurosurgery, Lahey Hospital & Medical Center – Beth Israel Lahey Health, UMass Chan Medical School, Boston, MA, USA

**Keywords:** Superficial siderosis, CSF leak, CSF-venous fistula, intracranial hypotension, vertigo, tinnitus

## Abstract

Superficial siderosis (SS) of the central nervous system is a rare chronic neurological disorder characterized by hemosiderin deposition over the subpial surface of the brain, cranial nerves, and spinal cord. This deposition can result from acute subarachnoid hemorrhage or from chronic or repeated hemorrhage, most often due to cerebral amyloid angiopathy and less commonly to cerebrospinal fluid (CSF) leaks, usually from ventral dural tears. Chronic microhemorrhages associated with spinal CSF leaks without ventral epidural CSF collections or meningoceles are exceedingly rare. Herewith, we describe a case of symptomatic SS as the sole clinical manifestation of a CSF-venous fistula (CSF-VF) of the thoracic spine. A male patient in his 60s presented with long-standing intermittent right-sided headache, anosmia, bilateral tinnitus, and gait instability. Neuraxis imaging revealed extensive SS involving the basal supratentorial brain, infratentorial brain, and spinal cord. A small intraforaminal thoracic nerve root dural ectasia was identified. There were no clear imaging signs of intracranial hypotension. Computed tomography myelography demonstrated a clear CSF-VF of the thoracic spine, which was subsequently closed by transvenous embolization. Postprocedure, the patient experienced progressive symptomatic improvement. This case highlights the importance of considering CSF-VF in the differential diagnosis of SS, especially when dural tears and epidural collections are absent on imaging.

## Introduction

Superficial siderosis (SS) of the central nervous system is a rare chronic neurological disorder characterized by hemosiderin deposition on the subpial layers of the brain, cranial nerves, and spinal cord. Superficial siderosis that does not result from a single inciting event such as trauma, aneurysmal subarachnoid hemorrhage, or surgery is usually associated with some form of recurrent, low-grade blood extravasation into the cerebrospinal fluid (CSF) space.^1–3^ Hemosiderin distribution in SS can be supra- or infratentorial or combined depending on the source of bleeding. Supratentorial SS is more common and usually secondary to cerebral amyloid angiopathy. Infratentorial SS is more rare and commonly associated with traumatic or spontaneous spinal CSF leaks, with or without symptomatic intracranial hypotension (SIH).^3–7^ In the vast majority of cases, these CSF leaks are ventral, with a large epidural collection. In a minority of cases, SS can be secondary to other types of CSF leaks, such as those seen in dural diverticula or CSF-venous fistulae (CSF-VF). From a clinical perspective, infratentorial SS causes a syndrome characterized by sensorineural hearing loss, cerebellar ataxia, and pyramidal signs. Rarer symptoms such as dementia, bladder disturbance, anosmia, anisocoria, extraocular motor palsies, neck or back pain, clumsiness, dysarthria, bilateral sciatica, and lower motor neuron signs have also been described.^1,6,8,9^ Herewith, we describe the case of a male patient in his 60s with symptomatic SS as the only symptom of a thoracic spine CSF-VF.

## Case report

A 64-year-old male with intermittent right-sided headache, anosmia, 18-month-long history of bilateral tinnitus, and gait instability was referred to our clinic for consideration for a cerebrospinal angiogram after magnetic resonance imaging (MRI) demonstrated significant SS of the entire neuraxis. Physical exam was notable for abnormal tandem gait, positive Romberg's sign, and upgoing plantar response. Laboratory tests, consisting of comprehensive sensory neuropathy panel, immunofixation serology, and serum copper levels were negative.

**Figure 1.**
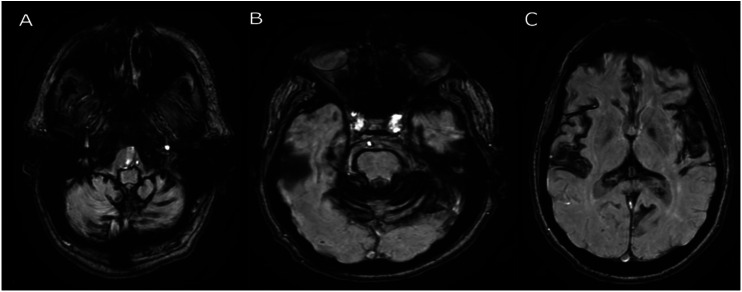
Susceptibility weighted imaging showing diffuse, prominent blooming artifact at the level of the brainstem (A), cerebellar folia and pons (B) and bilateral insular cortex (C), compatible with diffuse superficial siderosis.

**Figure 2.**
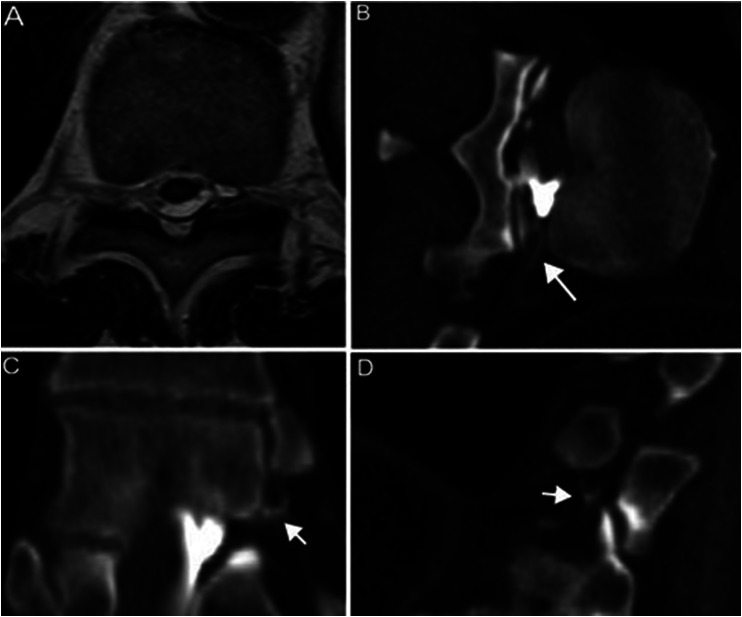
Heavily T2-weighted axial magnetic resonance imaging (MRI) demonstrating a small fluid collection in the medial portion of the left T10–T11 neural foramen (A), either compatible with a T10 nerve root dural ectasia or with a localized cerebrospinal fluid (CSF) leak. Left lateral decubitus computed tomography (CT) myelogram demonstrated a CSF-venous fistula at that level with drainage in the left T10 intersegmental vein (B, long arrow) and in multiple small periforaminal veins (C, D, short arrows).

Brain and spine MRI with and without contrast showed extensive SS on the pial surfaces of the inferior frontal and inferiomesial temporal lobes, brainstem, cerebellum, and cervical spinal cord. Superficial siderosis was evident, to a lesser extent, along with the entire length of the thoracolumbar cord (Figure 1). There were no intraparenchymal microhemorrhages and no clear findings of intracranial hypotension. On heavily T2 weighted high-resolution sequences of the spine, there was a prominent area of T2 hyperintensity in the medial segment of the left T10–T11 neural foramen compatible with a dural ectasia of the left T10 nerve root sleeve or with a localized CSF leak. Further diagnostic workup with bilateral decubitus computed tomography (CT) myelograms demonstrated a CSF-VF between a left T10 nerve root dural ectasia and the intersegmental vein at the same level (Figure 2). The patient was scheduled for transvenous embolization of the CSF-VF.

**Figure 3.**
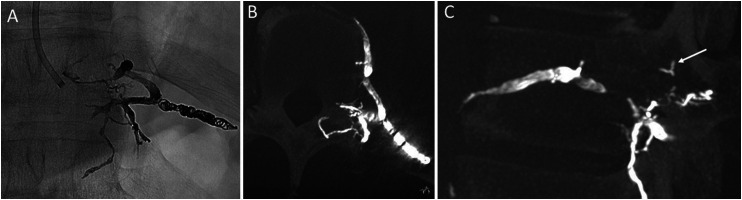
Post-embolization single-shot DSA acquisition (A) and cone-beam computed tomography (CT) images (B, C), demonstrating hyperdense embolic material in the draining T10–T11 intersegmental vein and in multiple periforaminal veins, including those visualized on the left lateral decubitus CT myelography (C, arrow).

Via a right transfemoral venous approach, a Benchmark guide catheter (Penumbra, Alameda, CA) was advanced into the hemiazygos vein and a 6 × 12 mm Eclipse dual lumen balloon microcatheter (Balt, Montmorency, France) was then navigated into the left T10 intersegmental vein. The proximal T10 intercostal division was preliminarily obliterated with Optiblock coils (Balt, Montmorency, France) to improve selectivity of the embolization. Next, the balloon was navigated into the radicular division of the vein and inflated. Onyx-34 (Medtronic, Minneapolis, MN) was then injected, distributing within the foraminal plexus and reaching the left lateral epidural compartment. Injection was continued during withdrawal of the deflated microballoon, further obliterating the mid portion of the T10 intersegmental vein. Distribution of the liquid embolic in the desired compartments was confirmed by a cone-beam CT performed in the neurointerventional suite (Figure 3).

The patient tolerated the procedure well and reported an improvement in headaches and gait instability within 3 weeks of discharge. At the latest follow-up, 9 months postprocedure, mild symptoms persisted but had stabilized. The patient was also undergoing chelation therapy for residual siderosis.

## Discussion

In this study, we report the case of a patient who had extensive, symptomatic SS of the brain, brainstem, and spinal cord as the sole symptom of a CSF-VF, without imaging stigmata of SIH. Superficial siderosis is a relatively rare chronic neurological disease, with a reported prevalence of 0.21% in individuals aged 50–69 years and 1.43% in those over 69 years. It has been divided into cortical and infratentorial types, with the denomination being based on the location of the most prominent hemosiderin deposition, although overlap between the two forms can be seen, as in our case. The cortical type is most commonly seen in the context of cerebral amyloid angiopathy.^10^ The infratentorial type can be associated with spinal CSF leaks secondary to trauma, surgery or brachial plexus avulsion, or that can be spontaneous. In large series of almost 100,000 MRIs obtained for all indications reviewed by Friedauer et al., SS related to CSF leaks was seen in 0.05 per 1000 brain MRI scans.^4^

Until recently, the only type of spontaneous CSF leak identified in association with SS was a ventral dural tear from osteophytes or calcified disc herniations. Such tears are associated with epidural CSF collections and are the most common causes of infratentorial SS, which is present in almost 10% of ventral dural leaks.^11^ The proposed mechanism for hemosiderin deposition in those cases is that recurrent trauma to the ventral spinal epidural plexus results in chronic microbleeding into the CSF space. Free CSF flow through the tear may also impede clot formation and bleeding cessation.^7^

The association between SS and other types of CSF leaks such as dural ectasias, nerve root diverticula, or CSF-VFs was first established in a recent series by Schievink et al. who analyzed a population of 1589 subjects with an established clinical and radiological diagnosis of SIH. Superficial siderosis was found in 3.6% (57) patients. Of these, 5 (9%) had a CSF-VF. In the subgroup of all patients with a CSF-VF, SS was present in 2.6%.^11^

Cerebrospinal fluid-venous fistulas were first described in 2014 and have since been increasingly recognized as an important cause of SIH without evident dural tears.^12^ They are commonly located on the anterior surface of dural ectasias of spinal nerve roots, at the normal site of CSF reabsorption, and contribute to around one-fourth of spontaneous intracranial hypotension cases. These lesions are diagnosed via dedicated decubitus digital subtraction or CT myelography and can be treated by transvenous endovascular embolization or surgical ligation, with excellent results.^13,14^ In SS due to a CSF-VFs, hemosiderin deposition may be due to the presence of intermittent bidirectional flow through the fistula with reflux of venous blood in the CSF space, or to microhemorrhages from traction of dural veins secondary to brain sagging.^12^ Unlike the cases described by Schievink, our patient did not meet imaging criteria for SIH. This case therefore constitutes, to the best of our knowledge, the first report of a CSF-VF exclusively discovered following a diagnosis of symptomatic SS. In keeping with other reports, the patient experienced symptomatic improvement after treatment.^15,16^

There are potentially impactful implications to the content of this report. Firstly, clinicians should be aware that CSF-VFs should be included in the differential diagnosis of SS, especially in the absence of an obvious ventral leak on dedicated spine imaging. Given the significant overlap between SS and SIH syndromes (tinnitus, ataxia, gait instability), it is possible that patients with MRI evidence of SS and absence of a dural tear have an undiagnosed CSF-VF but are not directed to the appropriate specialist given the absence of the classic imaging stigmata of SIH.^7^ This may result in a lengthy and frustrating diagnostic pathway, which may include unnecessary invasive examinations such as spinal angiograms, for which this patient was initially referred to us. It is known that classic imaging signs of SIH may have poor correlation with the severity of the clinical syndrome.^17^ A study which analyzed CSF samples of patients with proven SIH found that levels of ferritin and/or bilirubin were elevated in up to 30% of the patients, including some without imaging evidence of SS. The authors speculated on the existence of a preclinical phase of SS and the fact that microhemorrhages may be present in patients at risk of developing SS.^18^ It is therefore reasonable to include SS in the spectrum of manifestations of intracranial hypotension from CSF leaks.

Secondly, in Schievink's report, SS was present in only a small proportion (2.6%, 5/194) of the total of patients with CSF-VFs (194/1589, 12% of the total SIH cases). Being this an extremely selected population, it is possible that the true prevalence of CSF-VFs as cause of SS is higher than that. This assumption is corroborated by the fact that one patient with SS was found in group of seven asymptomatic patients with incidental radiological diagnosis of CSF-VFs, yielding a rate of 14%.^19^

Lastly, long-term prognosis in SS remains variable and depends largely on the duration of hemosiderin deposition and the extent of neuronal damage prior to intervention. Timely diagnosis and occlusion of the CSF-VFs may interrupt the chronic microbleeding and lead to halting of disease progression and various degrees of symptom relief as seen in our case and as reported by Schievink et al. and recently by El Rahal et al.^15,16^ Importantly, the latter report demonstrates how in order to maximize chances of symptom improvement, the treatment needs to be performed as early as possible in the course of the disease.

## Conclusion

This case underscores the need for heightened awareness of CSF-VFs as a potential etiology for SS, particularly in the absence of epidural CSF collections. Early recognition and intervention are crucial in preventing irreversible neurological deterioration.
